# Transcranial Low-Level Laser Therapy Improves Neurological Performance in Traumatic Brain Injury in Mice: Effect of Treatment Repetition Regimen

**DOI:** 10.1371/journal.pone.0053454

**Published:** 2013-01-07

**Authors:** Weijun Xuan, Fatma Vatansever, Liyi Huang, Qiuhe Wu, Yi Xuan, Tianhong Dai, Takahiro Ando, Tao Xu, Ying-Ying Huang, Michael R. Hamblin

**Affiliations:** 1 Wellman Center for Photomedicine, Massachusetts General Hospital, Boston, Massachusetts, United States of America; 2 Department of Dermatology, Harvard Medical School, Boston, Massachusetts, United States of America; 3 Department of Otolaryngology, Traditional Chinese Medical University of Guangxi, Nanning, China; 4 Department of Infectious Diseases, First Affiliated College & Hospital, Guangxi Medical University, Nanning, China; 5 Department of Burn, Jinan Center Hospital, Jinan, China; 6 School of Engineering, Tufts University, Medford, Massachusetts, United States of America; 7 Department of Electronics and Electrical Engineering, Keio University, Kohoku-ku, Yokohama, Japan; 8 Laboratory of Anesthesiology, Shanghai Jiaotong University, Shanghai, China; 9 Aesthetic and Plastic Center of Guangxi Medical University, Nanning, China; 10 Harvard-MIT Division of Health Sciences and Technology, Cambridge, Massachusetts, United States of America; University of South Florida, United States of America

## Abstract

Low-level laser (light) therapy (LLLT) has been clinically applied around the world for a spectrum of disorders requiring healing, regeneration and prevention of tissue death. One area that is attracting growing interest in this scope is the use of transcranial LLLT to treat stroke and traumatic brain injury (TBI). We developed a mouse model of severe TBI induced by controlled cortical impact and explored the effect of different treatment schedules. Adult male BALB/c mice were divided into 3 broad groups (a) sham-TBI sham-treatment, (b) real-TBI sham-treatment, and (c) real-TBI active-treatment. Mice received active-treatment (transcranial LLLT by continuous wave 810 nm laser, 25 mW/cm^2^, 18 J/cm^2^, spot diameter 1 cm) while sham-treatment was immobilization only, delivered either as a single treatment at 4 hours post TBI, as 3 daily treatments commencing at 4 hours post TBI or as 14 daily treatments. Mice were sacrificed at 0, 4, 7, 14 and 28 days post-TBI for histology or histomorphometry, and injected with bromodeoxyuridine (BrdU) at days 21–27 to allow identification of proliferating cells. Mice with severe TBI treated with 1-laser Tx (and to a greater extent 3-laser Tx) had significant improvements in neurological severity score (NSS), and wire-grip and motion test (WGMT). However 14-laser Tx provided no benefit over TBI-sham control. Mice receiving 1- and 3-laser Tx had smaller lesion size at 28-days (although the size increased over 4 weeks in all TBI-groups) and less Fluoro-Jade staining for degenerating neurons (at 14 days) than in TBI control and 14-laser Tx groups. There were more BrdU-positive cells in the lesion in 1- and 3-laser groups suggesting LLLT may increase neurogenesis. Transcranial NIR laser may provide benefit in cases of acute TBI provided the optimum treatment regimen is employed.

## Introduction

Incidences of traumatic brain injury (TBI) in both developed and developing countries are in rise. Main reasons that can account for that include growth in population and growing number of traffic accidents (and other emergencies such as natural disasters, sports injuries, falls and assaults. Moreover modern military conflict has led to an additional steep rise in TBI due to blast injuries as well as direct combat-mediated head injuries [1]. The burden of TBI in USA has been estimated to be 1.5 million cases each year, with an annual economic cost exceeding $56 billion [2].

The lack of any specifically approved therapy for TBI, combined with the failure of many clinical trials of pharmaceutical drugs that have been investigated for TBI, has motivated researchers to a widen their range in search of novel therapeutic interventions [3], [4]. These intervention avenues can be grouped into therapies that could potentially affect oxidative stress [5]; inflammation [6]; excitotoxicity [7]; metabolic dysfunction [8]; dysregulated neurochemical pathways [9]; impaired circulation [10]; brain hypoxia [11]; could increase neuroprotection [12] or stimulate neurogenesis [2] and could induce brain repair by stem cells [13]. Some physical intervention methods, such as brain hypothermia, have shown encouraging results [14].

Low level laser (light) therapy (LLLT), as a potential treatment avenue, has been clinically applied for a wide range of medical indications requiring protection from cell and tissue death, stimulation of healing and repair of injuries, and reduction of pain, swelling and inflammation [15]. Evidence is suggesting that red or near-infra-red light (at wavelengths that can penetrate tissue) is absorbed by mitochondrial chromophores leading to increased cellular respiration, more ATP synthesis, modulation of oxidative stress and nitric oxide production that together lead to activation of signaling pathways and gene transcription [16]. One area that is attracting growing interest is the use of transcranial LLLT to treat stroke [17], [18] the success of which has been demonstrated both in animal models [19] and in clinical trials [20]. To date, seven published studies on mouse models [21], [22], [23], [24], [25], [26], [27] have suggested that transcranial LLLT (810 nm laser could have a beneficial therapeutic effect on TBI as well. However there are still many questions to be answered, for instance, what is the best regimen of treatment repetition? One observation that has been repeatedly made, during the 40 years of LLLT studies, is that there is a pervasive biphasic dose response relationship that applies not only in cell culture (*in vitro*) studies, but also in pre-clinical (*in vivo*) animal studies, and even in clinical reports [28]. It has been found that there is generally an optimum level of energy density (J/cm^2^), power density (mW/cm^2^) and/or treatment repetition to give the best therapeutic effects and if the optimum parameter setting is either not reached or else is substantially exceeded the benefit is reduced [29]. In our current study we used a controlled cortical impact (CCI) mouse model of severe TBI, and observed the effects of different treatment repetitions of 810 nm LLLT on neurobehavioral and vestibulomotor functioning; followed the histomorphological analyses, and histological evidence to track the neuroprotection and neurogenesis.

## Materials and Methods

### 2.1 Animals

#### Ethics statement

All animal procedures were approved by the Subcommittee on Research Animal Care (IACUC) of the Massachusetts General Hospital (protocol # 2010N000202) and met the guidelines of the National Institutes of Health.

Adult male BALB/c mice (6–8 weeks, weight 21 to 25 g; Charles River Laboratories, Wilmington, MA) were used in the study. The mice were divided into 9 groups: 3 sham-TBI sham-treatment groups, 3 real-TBI sham-treatment groups, and 3 real-TBI active-treatment groups (see later); each group had 16 mice at the start of the study, allowing for sacrifice of 2mice at time points and allowing 8mice to remain between 14 and 28 days. The animals were housed with one mouse per cage and were maintained on a 12 h light &12 h dark cycle with access to food and water ad libitem.

### 2.2 Mouse Model of Controlled Cortical Impact Focal (TBI)

The mice were anesthetized with isoflurane for the surgical procedure. After the hair on the head was shaved and depilated (Nair, Carter-Wallace, NY), the top of the skull was adequately exposed with a 1 cm skin incision made in a central and sagittal direction. A 5 mm craniotomy was performed over the right parietotemporal cortex using a trephine attached to an electric portable drill taking care to avoid damaging the meninges. The bone flap was removed and mice were subjected to controlled cortical impact using a pneumatic impact device (Model AMS 201, AmScien Instruments LLC, USA) with a 3 mm flat-tip, high pressure 150 psi, low pressure 30 psi, rod speed 4.8 m/sec, rising duration 8.41 ms, and set impact depth of 2 mm with the devise positioned over the right front-parietal cortex and the tip centered 3 mm anterior to lambda and 2.5 mm right of midline within the craniotomy; these parameters were selected to yield a trauma giving a neurological severity score (NSS) of 7–8 measured 1 hour post-TBI. Immediately after generating the brain trauma the craniotomy hole was sealed with bone wax, the scalp incision was closed with sutures. Sham control mice were subjected to all aspects of the protocol (anesthesia, skin incision, and craniotomy) except for cortical impact. After recovery from anesthesia, the mice were returned to their cages with postoperative care and ad libitem access to food and water. Mice which had NSS scores greater than 8 or less than 7 were excluded from the study.

### 2.3 Neurobehavioral Testing

The neurological status of the traumatized mice was evaluated at different time intervals after CCI according to NSS (Table 1). The neurological tests are based on the ability of the mice to perform 10 different tasks [24] that evaluate the motor ability, balance, and alertness of the mouse. One point is given for failing to perform each of the tasks; thus, a normal, uninjured mouse scores 0 and the maximum is 10. The severity of injury is defined by the initial NSS, evaluated 1 h post-CCI, and is a reliable predictor of the late outcome. Thus, very severe injury is defined in mice having an NSS of 9–10; severe injury in mice with an NSS of 7–8; moderate injury with NSS of 5–6, and mild injury in mice with an NSS of <5. NSS was carried out on days 0, 1, 4, 7, 10, 15, 19, 24, and 28.

**Table 1:** Neurological Severity Score (NSS) for TBI Mice.

Task	NSS
Presence of mono- or hemiparesis	1
Inability to walk on a 3-cm-wide beam	1
Inability to walk on a 2-cm-wide beam	1
Inability to walk on a 1-cm-wide beam	1
Inability to balance on a 1-cm-wide beam	1
Inability to balance on a round stick (0.5 cm wide)	1
Failure to exit a 30-cm-diameter circle (for 2 min)	1
Inability to walk straight	1
Loss of startle behavior	1
Loss of seeking behavior	1
Maximum total	10

Mice are awarded 1 point for each failure to complete a task.

Motor function or muscle power was assessed using a wire grip and motion test (WGMT), which was developed on the basis of the test of gross vestibulomotor function [30]. The test consisted of placing the mouse on a thin and horizontal metal wire (45 cm long) suspended (45 cm above a foam pad) between two poles, and grading the ability of the mouse to grip, attach and move as described in Table 2. The wire grip test was performed in triplicate and an average value calculated for each mouse. WGMT was carried out on days 0, 1, 4, 7, 10, 15, 19, 24, and 28.

**Table 2:** Wire grip and motion score for TBI mice.

Task	score
Unable to grasp wire for 30 sec	0
Gripping wire for 60 sec with 1 or 2 paws	1
Jump up and grasp wire with 2 paws	2
Grasp wire with 4 paws and wrap tail around	3
Crawl along the wire for at least 1 inch	4
Crawl along wire to terminal and dismount	5
Maximum total	5

Mice are assessed the score for the best level they reach.

### 2.4 Laser Treatment

Mice were lightly anesthetized with isoflurane and immobilized by taping their paws to a plastic plate. Three groups of TBI mice received active laser treatment with 810 nm continuous wave laser (DioDent Micro 810, HOYA ConBio, Fremont, CA) using a fluence of 18 J/cm^2^ at an irradiance of 25 mW/cm^2^ taking 12 minutes with a spot size 1 cm diameter positioned centrally on top of the mouse head and delivered 4 hours post-injury. Group 1 received a single laser Tx, Group 2 received laser treatments on days 1, 2, and 3. Group 3 received fourteen daily laser Tx (days 1–14). There were 3 sham-TBI control groups of mice that received craniotomy but no CCI (group 4 receiving a single anesthesia and taping, group 5 received 3 daily sessions of anesthesia and taping and group 6 received 14 daily sessions of anesthesia and taping). Likewise there were 3 real-TBI sham-treated control groups that received TBI followed by 1 (group 7), 3 (group 8), and 14 (group 9) sessions of anesthesia and taping respectively.

### 2.5 Histomorphology

At the end of the appropriate follow-up period; 4 hours, 4 days, 1 week, 2 weeks, and 4 weeks post TBI, 2 mice per group were randomly chosen and deeply anesthetized with isoflurane and subsequently perfused transcardially with 0.9% saline, followed by 4% phosphate-buffered paraformaldehyde. Brains were then removed from the skull and photographed. All specimens were fixed in the same formaldehyde solution for 3 days and embedded in paraffin. 5-mm thick cross-sectional coronal slices were taken including the injured region of the brain; and 10 micron-thick sections were cut from the top, middle, and bottom of the thick slice block by microtome. These 10-micron sections were used for hematoxylin-eosin (H&E) and Fluoro-Jade staining, while 5 micron-thick coronal sections were taken for BrdU staining. The brain sections stained with H&E were pictured by using AutoPix (automated laser capture micro-dissection). The brain lesion volume was then calculated by multiplying the average lesion area of the three sections by the thickness of the slices. Fractional lesion volume is the ratio of difference between non-traumatized brain volume and hemisphere volume to traumatized lesion volume.

### 2.6 BrdU Labeling by Immunohistochemistry

Before sacrifice at 28 days mice (6 per group) were given seven consecutive daily intraperitoneal injections of 5-bromo-2′-deoxyuridine (BrdU) (Sigma, St Louis, MO) as 50 mg/kg dilution in saline. Briefly, the unstained paraffin slices were respectively immersed in xylene for deparaffinization, graded ethanol for rehydration, and then passed through antigen retrieval with citrate buffer solution in microwave-oven, incubated in blocking solution consisting of 5%BSA/0.1%TritonX-100 in PBS, and immunostained with immunostained with the rat anti-BrdU (Abcam, Cambridge, MA) at 1∶50 working concentration. Selected goat anti-rat secondary antibodies matched with primary antibodies (Alexa-fluor 594-conjugated, Invitrogen) to stain at a 1∶500 concentration. Finally, they were cover-slipped with mounting media contained DAPI (Fisher Scientific). The BrdU positive cells were imaged with a confocal microscope (Olympus America Inc, Center Valley, PA). Red BrdU staining was quantified by the use of Image J.

### 2.7 Fluoro-Jade C Staining

The 10 micron-thick slices were first deparaffinized in a slice warmer, and then respectively immersed in 100% ethanol, a basic alcohol solution consisting of 1% NaOH in 80% ethanol, followed by a wash in 70% ethanol. The tissue was briefly rinsed with distilled water and incubated in 0.06% KMnO_4_ solution, afterwards, the slices were transferred to a 0.001% solution of Fluoro-Jade C (Millipore) dissolved in 0.1% acetic acid. The slices were rinsed through three changes of distilled water. The air-dried slices were cleared in xylene and then cover-slipped with DAPI as above. Green Fluoro-Jade C staining was quantified by the use of Image J.

### 2.8 Statistics

Lesion size and labeled cells were measured with Image J software. Mice numbers were n = 8–16 per group, depending on how many had been sacrificed. In a two-dimensional coordinate system, the area-under-the-curve (AUC) data, which represent the time courses of NSS or WGMT in the various groups of mice, were calculated using numerical integration [31]. Data are presented as mean ± SD, and statistically analyzed using one-way analysis of variance (ANOVA) followed by Tukey post-hoc test for multiple comparisons. Significance was defined as p<0.05. SPSS statistics V17.0 software was used for statistical analyses.

## Results

### 3.1 Neurobehavioral Evaluation

**Figure 1:**
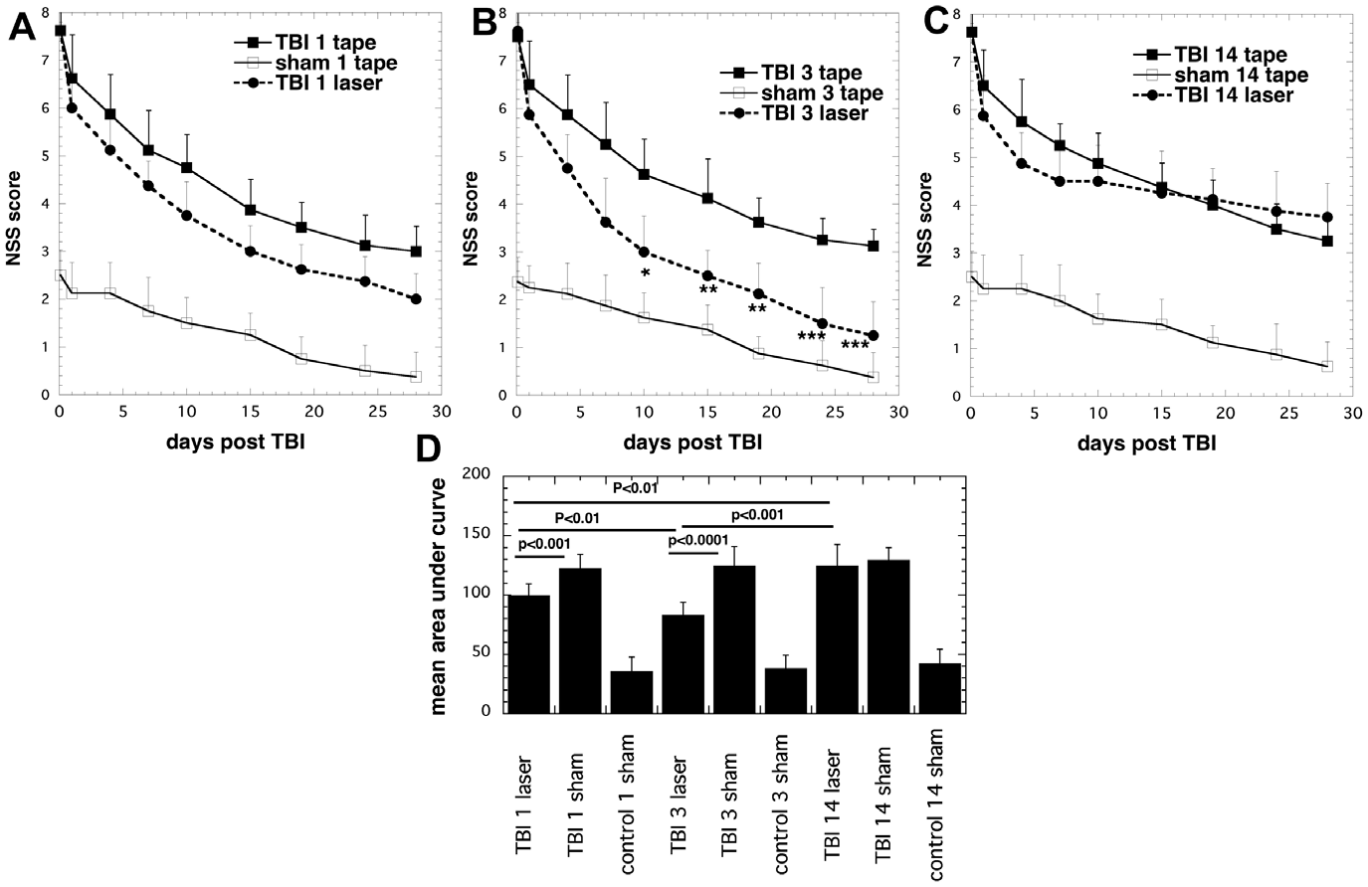
NSS scores of the mice. Mean (n = 8–14) NSS scores measured over 4 weeks of mice in 9 groups consisting of sham TBI mice, sham control mice, 810-nm laser treated TBI mice (18 J/cm^2^ delivered at 25 mW/cm^2^). (A) Mice and controls given 1 laser Tx or 1 sham Tx at 4 hours post TBI; (B) Mice and controls given 3 daily laser Tx or 3 sham Tx; (C) Mice and controls given 14 daily laser Tx or 14 sham Tx. * p<0.05; ** P<0.01; *** p<0.001. One way ANOVA. (D) Mean areas under curve of NSS time courses from mice in different groups. P values given were determined by one way ANOVA.

The neurological behavior was evaluated by NSS (Fig. 1A–1C). The deficits in neurobehavioral function in all groups of mice with severe TBI gradually but steadily improved as time passed after the induction of TBI. However, we observed greater improvements in group 1 (single laser Tx given at 4hours post TBI; Fig. 1A) compared to group 7 (real-TBI, one immobilization and no laser) that became especially noticeable as time progressed up to 28 days post-TBI. The improvement seen in group 2 that received three laser treatments on days 1–3 post-TBI was even more pronounced and statistically significant (P<0.05) when compared to control group 8 (real-TBI 3-sham Tx; Fig. 1B). Group 3 that received 14-daily laser Tx; Fig. 1C) showed improvement in NSS until day 5 at which point the mice had received 5 daily laser Tx. However, the improvement then ceased, as more laser Tx were given, and at day 14, the advantage over group 9 (real-TBI 14 sham Tx) had disappeared. At day 28 there was no difference compared to TBI-sham Tx group 9. Figure 1D shows the integrated time courses of the NSS scores. The values for mice with real-TBI and receiving both 1 laser Tx and 3 laser Tx were significantly better (P<0.001) then the corresponding real-TBI sham Tx groups. Furthermore the values for real-TBI 3 active laser Tx was significantly better (P<0.01) than real-TBI 1 active laser Tx. The integrated NSS value of the group of real-TBI 14 active laser Tx was significantly worse than the group of real-TBI 3 active laser Tx (P<0.001) and also worse that the group of real-TBI 1 active laser Tx (P<0.01).

### 3.2. Muscular and Vestibulomotor Evaluation

**Figure 2:**
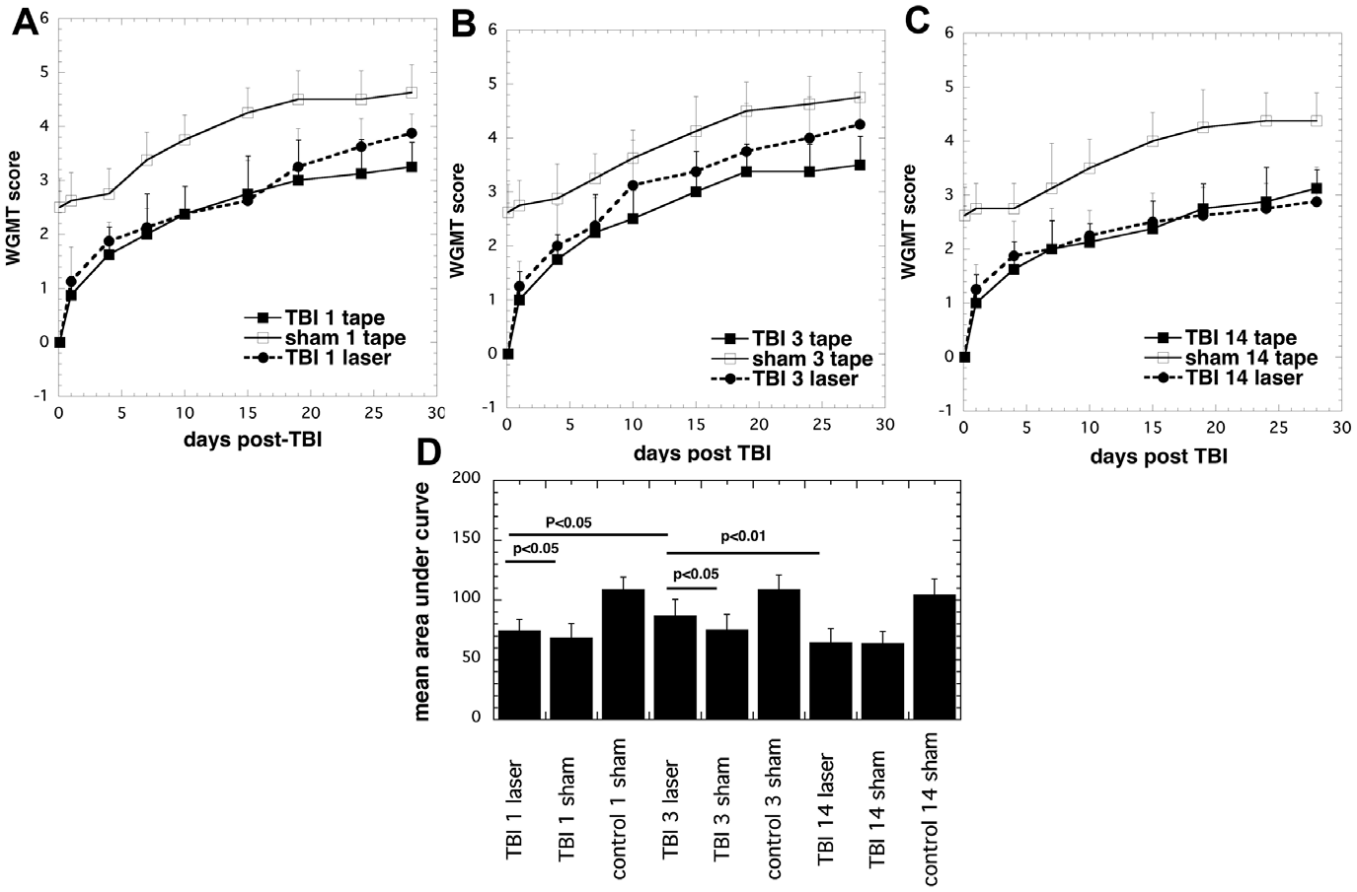
WGMT scores of the mice. Mean (n = 8–14) WGMT scores measured over 4 weeks, of mice in 9 groups consisting of sham TBI mice, sham control mice, 810-nm laser treated TBI mice (18 J/cm^2^ delivered at 25 mW/cm^2^). (A) Mice and controls given 1 laser Tx or 1 sham Tx at 4 hours post TBI; (B) Mice and controls given 3 daily laser Tx or 3 sham Tx; (C) Mice and controls given 14 daily laser Tx or 14 sham Tx. (D) Mean areas under curve of WGMT time courses from mice in different groups. P values given were determined by one-way ANOVA.

The results for muscle power and motor function were evaluated by WGMT (Fig. 2A–2C). There were similar overall findings to those found for the NSS. Group 1 (1 laser Tx; Fig. 2A) improved compared to real-TBI sham-Tx group 7. Group 2 (3 laser Tx; Fig. 2B) showed a greater improvement over real-TBI 3 sham-Tx group 8, while group 3 (14 laser Tx; Fig. 2C) showed no improvement over real-TBI sham-Tx group 9 at day 28. Figure 2D shows the integrated time courses of the WGMT scores. The values for mice with real-TBI and receiving both 1 laser Tx and 3 laser Tx were significantly better (P<0.05) then the corresponding real-TBI sham Tx groups. Furthermore the values for real-TBI 3 active laser Tx was significantly better (P<0.05) than real-TBI 1 active laser Tx. The integrated WGMT value of the group of real-TBI 14 active laser Tx was significantly worse than the group of real-TBI 3 active laser Tx (P<0.01) and but not different from the group of real-TBI 1 active laser Tx.

### 3.3. Size of Brain Lesion by Histomorphometry

**Figure 3:**
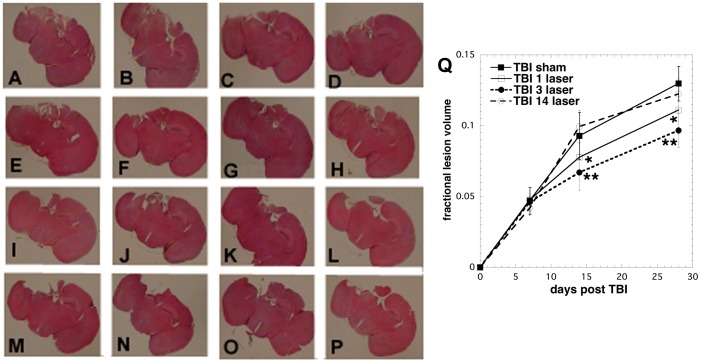
Brain lesion size in the mice. Example whole brain cross-sections taken from mice sacrificed at day 4 (A–D), day 7 (E–H), day 14 (I–L) and day 28 (M–P). Mice were taken from TBI sham Tx group (A, E, I, M); TBI 1 laser Tx group (B, F, J, N); TBI 3 laser Tx group (C, G, K, O); TBI 14 laser Tx group (D, H, L, P). (Q) Mean (n = 2–3) brain fractional lesion volumes calculated as described at 1, 2, and 4 weeks in brains of mice in 4 groups consisting of sham TBI mice, 810-nm laser treated TBI mice (18 J/cm^2^ delivered at 25 mW/cm^2^ given 1, 3 or 14 laser Tx. ** P<0.01; * p<0.05 vs TBI sham and TBI 14 laser Tx groups. One way ANOVA.

Examples of H&E stained brain slices taken from mice in the various groups are shown in Fig. 3A–P. All groups showed an expansion of the lesion size in the brain over the course of 4 weeks. This finding suggested that the lesion is developing (expanding) in size at the same time as the functional NSS and WGMT results are improving. The quantitative results of the histomorphometry measurements of the brain lesion volume from mice sacrificed at 1, 2 and 4 weeks post-TBI are shown in Fig. 3Q. There was no difference in lesion volume between any of the groups at 7 days post-TBI. The mean lesion size of group 2 (3 Tx LLLT) or group 1 (1 Tx LLLT) was smaller than other TBI control groups at both 14 and 28 days (P<0.01 or P<0.05), while the result of the 3-time-LLLT group was better than that of the single laser Tx. The mean lesion volume of the mice that received 14 laser Tx was not significantly different from real-TBI sham-Tx control at any time point. These data are in agreement with the previous neurobehavioral evaluations (NSS and WGMT).

### 3.4. Fluoro-Jade C Staining

**Figure 4:**
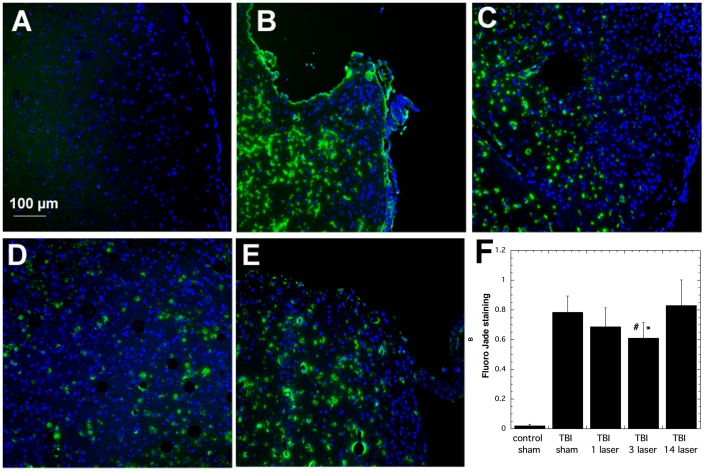
Fluoro Jade C staining of lesion areas in brain sections. All sections were taken from mice sacrificed at 14 days post-TBI. Green is FJC and blue is DAPI )control for equal numbers of cell nuclei. (A) Sham-TBI control mouse; (B) real-TBI sham Tx mouse. (C) TBI mouse treated with 1 laser Tx. (C) TBI mouse treated with 3 laser Tx (E) TBI mouse treated with 14 laser Tx (F) Mean green staining calculated with Image J from mice (n = 2–3) in the groups in panels A–E. # P<0.05 vs TBI sham; * P<0.05 vs 14 laser Tx. One way ANOVA.

Fluoro Jade C staining is used as a specific marker of degenerating neurons. In this study it was carried out at day 14 in the lesion area. The results from Fluoro-Jade C staining at day 14 showed (Fig. 4) that sham-TBI control mice (Fig. 4A) had virtually none as expected, there was robust staining in real-TBI sham Tx control mice (Fig. 4B), much less staining in real-TBI mice with 1 laser Tx (Fig. 4C), even less in real-TBI mice treated with 3 laser Tx (Fig. 4D), but noticeably more Fluoro Jade staining in real-TBI mice treated with 14 laser Tx (Fig. 4E). The mean Fluoro-Jade staining from 8 slides taken from 2–3 mice per group is shown in Fig. 4F and the staining in the 3 laser Tx group was significantly lower (P<0.05) compared to that in the real-TBI sham Tx group and the real-TBI mice treated with 14 laser Tx.

### 3.5. BrdU Staining

**Figure 5:**
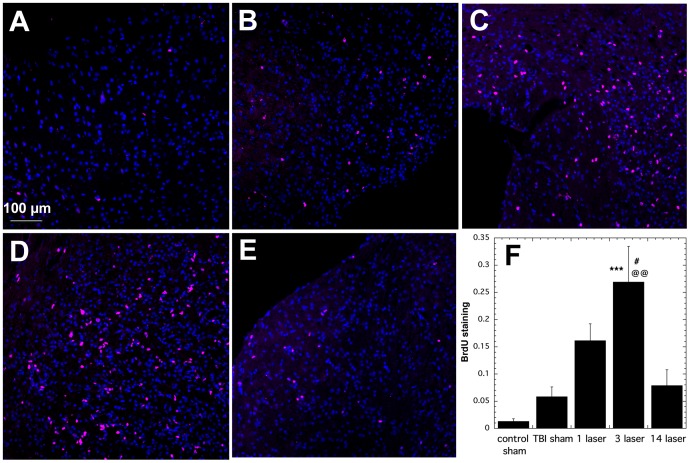
BrdU staining of lesion areas in brain sections. All sections were taken from mice sacrificed at 28 days post-TBI. Red is BrdU and blue is DAPI (control for equal numbers of cell nuclei). (A) Sham control mouse; (B) TBI sham untreated mouse. (C) TBI mouse treated with 1 laser Tx. (C) TBI mouse treated with 3 laser Tx (E) TBI mouse treated with 14 laser Tx (F) Mean (n = 6) red staining calculated with Image J from mice (n = 6) in the groups in panels A–E. *** P<0.001 vs TBI sham; # P<0.05 vs 1 laser Tx; @@ P<0.01 vs 14 laser Tx. One way ANOVA.

BrdU staining is used as a marker for proliferating cells as the compound is injected systemically for several days sacrifice and is incorporated into the DNA of dividing cells. Its presence can be readily detected by immunostaining. Effects of different regiments of LLLT on levels of BrdU at day 28 are shown in Fig. 5. There were only scattered BrdU+ cells present in sham-control mice, (Fig. 5A) more were seen in TBI-sham untreated mice (Fig. 5B), and many more in TBI mice that received 1 laser Tx (Fig. 5C). Mice with 3 laser Tx had even more BrdU+ cells (Fig. 5D) while there was a sharp drop seen in mice that received 14 laser Tx (Fig. 5E). The quantification shown in Fig. 5F indicates that the BrdU staining in the 3-laser Tx group was significantly higher than real-TBI sham Tx group (P<0.001), real-TBI 1 laser group (P<0.05) and the real-TBI 14 laser group (P<0.01).

## Discussion

This study has shown marked improvement in several parameters related to neurological, motor and lesion size in mice subjected to severe CCI-TBI and treated with 1 or 3 (but not 14) transcranial LLLT treatments. The mechanism appears to involve both prevention of tissue damage in the short term and increased brain repair in the longer term due to neurogenesis.

Serious TBI in the acute phase includes skull fracture, acute cerebral vascular damage, primary mechanical injury to the brain tissue, and these traumatic events lead to a series of molecular and cellular reactions that cause secondary brain injury. Once the brain is damaged, even a very short period of ischemia can cause significant physiological changes. Mechanical brain injury causes partial or whole brain blood flow decline, followed by lack of oxygen and glucose supplies to the neurons. If this situation is prolonged, it leads to neuronal cell death. Owing to local cerebral ischemia, cells surrounding the injured region are more sensitive to pathological changes, and therefore are liable to die in the succeeding days and weeks after the injury. This area is also known as the penumbra zone [32].

The brain slices in our study showed that the lesion size or cavity formation post-TBI underwent a gradual increase in size, developing from relatively shallow to big and deep, suggesting that these mice underwent a series of pathological changes of ischemia, hypoxia, metabolic disorder, edema, necrosis, infarction, collapse, and tissue absorption. We found that although local lesion sizes post-TBI become larger gradually over 1 month, contrarily, the neurobehavioral function and motor function in TBI mice progressively improved over the same period. This observation may be possibly explained by the phenomenon of neuroplasticity, a process whereby distant areas of the brain can adapt to take over the functions of damaged areas of the brain. In the present case it is possible that the contralateral cerebral cortex can gradually take over the functions previously undertaken by the damaged cortex.

The lesion size at both 14 and 28 days in mice treated with 1 or 3 LLLT Tx was significantly smaller than that in TBI control groups and also less than that seen in mice that received 14 LLLT Tx. This suggests that the laser is preventing or slowing down the development of the secondary brain injury.

The results of the performance assays, both NSS and WGMT, indicated that an optimized LLLT repetition regimen would produce the optimum effect on neurological, cognitive and motor function post-TBI. In every case 1-laser Tx was effective compared to the appropriate sham treated control, while the effect of 3-laser Tx was best, and excessively repeated LLLT (14 laser Tx) had lost all its beneficial effect. However, interestingly, even though the effect of 14 laser Tx at 18 J/cm^2^ was not as good as other laser groups, it was not significantly different in comparison with TBI control group, suggesting that our transcranial LLLT approach was relatively safe. The ineffectiveness of excessively repeated applications of LLLT was not surprising. There are two reviews [28], [29] that have collected all the examples of the biphasic dose response effect in LLLT. LLLT regimens that are based on too high a fluence (J/cm^2^), too high an irradiance (mW/cm^2^), or repeated too often can all have markedly inferior effects to regimens based on lower doses of LLLT. This biphasic dose response has been observed in cell culture, in animal studies and in clinical cases and trials of LLLT alike.

Previous research [33] showed that TBI induces cognitive deficit. Moreover, TBI-induced cognitive impairment was related closely with hippocampal regional excitability or neuronal loss. Fluid percussion TBI in mice results in significant hippocampal-dependent cognitive impairment, specifically retrograde amnesia. In a mouse model of pilocarpine-induced status epilepticus, hippocampal impairment was found to lead cognitive alterations, increased anxiety-like behavior, decreased depression-like behavior and impaired spatial learning and memory [34].

There have been five previous reports of transcranial LLLT having beneficial effects in mouse models of TBI. The first by Oron et al. [24] used a closed head weight drop model and found a single application of 808 nm transcranial laser improved NSS and reduced lesion size. A study by Ando et al [23] employed a CCI model and compared single applications of pulsed wave (PW) and CW 810 nm transcranial laser. They found that 10 Hz PW was superior to CW and 100 Hz PW in improving NSS, reducing both lesion size and depressive symptoms as measured by forced swim test and tail suspension test. A report by Khuman et al [22] compared a single application of 810 nm laser delivered transcranially or directly to the contused brain through the craniotomy in CCI. They found the direct LLLT improved Morris Water Maze performance and reduced microgliosis. A second study from Oron et al [21] in their weight drop model compared a single application of CW and 100 Hz PW 810 nm laser delivered 4, 6, or 8 hours post-TBI. The PW was best at improving NSS and the 8 h time delay was ineffective. A recent report from Oron et al [21] compared four different wavelengths (660 nm, 730 nm, 810 nm and 980 nm) of a single exposure to transcranial laser 4 h post a weight drop TBI. Both the 660 nm and the 810 nm (but not 730 nm or 980 nm) were able to improve the NSS score.

The cellular and molecular mechanisms of LLLT are beginning to be understood [15]. Red and near-infrared photons are absorbed in the mitochondria by chromophores such as cytochrome c oxidase [35]. Increased mitochondrial membrane potential, increased ATP production [36], modulation of reactive oxygen species [16], nitric oxide release [37], intracellular calcium [38] then follow. Signaling pathways and transcription factors are activated, leading to production of anti-apoptotic, pro-proliferation, antioxidant, anti-inflammatory and proangiogenic factors [28]. How these cellular events precisely affect neuroprotection and neurorepair remains to be elucidated. There have been numerous studies on transcranial laser therapy (TLT) using 810 nm lasers for stroke [39]. In animal models of stroke, it has been shown that TLT can improve neurological performance after occlusion of middle cerebral artery in rats [40] and embolic stroke in rabbits [41]. The noted success of the animal studies instigated clinical trials of TLT in human patients [17]. The first study (NEST-1) was successful [42], while the second failed to meet its primary endpoint [43] but a subsequent sub-analysis found significant improvement in a sub-set of patients [20]. There has been one clinical case report [44] of transcranial red/NIR (633 nm and 870 nm) LED therapy in two chronic TBI patients giving marked improvement in cognitive function after treatment once a week for eight weeks.

In the light of accumulated thus far data it was imperative for us to determine if LLLT could activate brain repair pathways, since until relatively recently it was thought that the adult brain was incapable of repairing itself [45]. However since the discovery of adult neurogenesis, originating in the dentate gyrus of the hippocampus and in the sub-ventricular zone of the lateral ventricle, this view has been overturned [46]. It is now thought that neuroprogenitor cells can be stimulated, especially in response to brain injuries suffered as a result of stroke or TBI, and that these cells may be able to migrate to the sites of brain damage to effect repairs [47]. BrdU is a synthetic thymidine analog that gets incorporated into cellular DNA when the cell is dividing (during the S-phase of the cell cycle), therefore, it is commonly used to detect proliferating cells in living tissues [48]. In our current study we discovered that the correct regimen of LLLT (1 or 3 daily laser Tx) could induce abundant BrdU positive cells in the lesion region at day 28 post-TBI, while the sham treated TBI control group hardly ever showed BrdU+ cells in the lesion region. It is important to point out that these cells are capable of generating new mature neurons in the pericontusional cortex and striatum [49], [50], [51], so if our future research can confirm that LLLT can increase neurogenesis in the damaged brain (or even in the normal brain), the number of possible applications of transcranial LLLT will increase dramatically.

The neuropathological study of Schmued, et al. [52] showed that Fluoro-Jade C, an anionic dye, can stain all degenerating neurons, regardless of specific insult or mechanism of cell death with high fidelity and resolution. However Fluoro-Jade C labeling showed greater morphological detail of degenerating neurons than its predecessors, Fluoro-Jade or Fluoro-Jade B, and was the most sensitive of the fluorescent markers for neuronal degeneration, in terms of producing an image of highest resolution and contrast [53], [54]. The ability to label with increased resolution implies that Fluoro-Jade C possesses a higher affinity for the endogenous neurodegeneration molecule(s) than its predecessors Fluoro-Jade or Fluoro-Jade B. Our research showed that the correct regimen of LLLT (3 or 1 laser Tx) could reduce the Fluoro-Jade C labeling of cortical neurons, moreover, there was a significant difference in comparison with TBI control group or excessive 14X-LLLT.

The study reported here have the following caveats: double staining for BrdU and the mature neuronal marker NeuN would have confirmed that the BrdU positive were newly formed neurons rather than proliferating glial cells or even delayed neuronal apoptosis [55]. We only carried out BrdU injection before sacrifice at the 28^th^ day time point. In order to obtain a better overall picture of the time course of the neurogenesis BrdU should be administered at earlier (and possible even later) time points.

In conclusion, our initial research indicates that transcranial laser is a promising treatment for TBI in mice. However, selecting a proper LLLT regimen has shown to be a key factor for optimal therapeutic effect. Our data demonstrated 3-time LLLT possessed a better effect on severe TBI in mice than other regimens and that 14-laser Tx had no benefit at 14 or at 28 days. Moreover, our results can append to the effects of LLLT for TBI in mice where it could significantly improve neural function, decrease lesion volume, augment cell proliferation, and even protect the brain against neuronal damage to some degree. Further studies will concentrate on the effects of LLLT on the hippocampus and subventricular zone regions of the brain in the TBI mice as these are considered to be the principal source of the neuroprogenitor cells. Our current study results combined with accumulated thus far *in vivo* and clinical study data are providing compelling evidence that LLLT can be a promising intervention avenue in treatment of neurological impairments and it is worth pursuing with in depth clinical trials.
